# The effects of regular training on spinal posture: a fitness and bodybuilding perspective

**DOI:** 10.3389/fphys.2025.1559150

**Published:** 2025-03-05

**Authors:** Mustafa Bingöl, Şaban Ünver, Hakkı Mor, Yunus Berk, Tülay Ceylan, Deniz Günay Derebaşı, Yağmur Naz Aydın, Tülin Akman, Hamza Küçük, Levent Ceylan

**Affiliations:** ^1^ Department of Physical Education and Sports Teaching, Faculty of Sports Sciences, Yüzüncü Yıl University, Van, Türkiye; ^2^ Department of Coaching Education, Yaşar Doğu Faculty of Sports Sciences, Ondokuz Mayıs University, Samsun, Türkiye; ^3^ Department of Coaching Education, Faculty of Sports Sciences, Yüzüncü Yıl University, Van, Türkiye; ^4^ Department of Physical Education and Sports Teaching, Graduate School, Ondokuz Mayıs University, Samsun, Türkiye; ^5^ Department of Physical Education and Sports Teaching, Yaşar Doğu Faculty of Sports Sciences, Ondokuz Mayıs University, Samsun, Türkiye; ^6^ Department of Coaching Education, Graduate School, Ondokuz Mayıs University, Samsun, Türkiye; ^7^ Department of Sport Management, Faculty of Sports Sciences, Hitit University, Çorum, Türkiye

**Keywords:** spinal posture, regular training, spinal cord, fitness training, physical activity

## Abstract

**Background:**

Regular training is associated with better postural alignment, decreased risk of musculoskeletal problems, and improved overall physical function. The aim of this study was to examine the changes in the spinal postures of individuals who regularly engage in fitness and bodybuilding training.

**Methods:**

A total of 252 male individuals between the ages of 20–28 (mean age: 23.71 ± 1.81 years), who either engage in fitness and bodybuilding training (athletes) (n = 125; age 23.61 ± 1.78 years; sports age 5 ± 0.89 years) and those who do not engage in sports (sedentary) (n = 127; age 23.82 ± 1.83 years) participated in the study. A spinal Mouse device was used in the measurements. In spine measurement, all spinal protrusions from C7 to S1 were evaluated. T-test and correlation tests were used to analyze the data.

**Results:**

A statistically significant difference was detected between the athlete and sedentary groups regarding the degrees of thoracic kyphosis, lumbar lordosis, and sacral kyphosis (p < 0.05). However, no difference was observed in the upright posture (p > 0.05).

**Conclusion:**

It was found that there were significant differences in some spinal curves of fitness and bodybuilding trainees compared to sedentary, but the values were within physiological limits and did not indicate negative effects. The study may provide new insights into the effects of fitness and bodybuilding training on spine health, and individuals can incorporate training with proper form and technique into their lifestyle for spinal health.

## 1 Introduction

Participation in sports is considered one of the fundamental conditions for a healthy and balanced life (Kürkçü and Gökhan, 2021). Individuals who exercise regularly and consistently live more energetic and positive social lives than sedentary individuals (Biddle et al., 2019) In a healthy individual’s standing posture, the spine has four natural curves: cervical lordosis, thoracic kyphosis, lumbar lordosis, and sacral kyphosis, and these curves are biomechanically interrelated (Lau et al., 2010). Maintaining these natural spinal curvatures contributes to the overall health of individuals, while regular physical activity positively affects spinal health (Miao, 2024). Also, it has been well-established that resistance training is an important strategy for treating and preventing many diseases (Figueiredo et al., 2018; Arslanoglu et al., 2024; Kucuk et al., 2024; Ünver et al., 2024; Arslan et al., 2024; Morenas-Aguilar et al., 2025).

Resistance training encompasses a variety of modalities. Among these, fitness and bodybuilding training appear to offer a multitude of benefits. Specifically, fitness training has been shown to facilitate fat burning, reduce fat mass, and increase muscle mass while protecting against injury. Therefore, increasing lean body mass is a primary goal for individuals engaged in weight training (Schoenfeld, 2010). The skeletal muscles account for approximately 35% of the total body weight, and the muscles, an important part of the musculoskeletal system, play an important role in glucose metabolism, endocrine function, and thermogenesis. With respect to the demographic changes in Western societies, the protection of muscle mass is of utmost importance. It has been well-documented that increased age is associated with a loss of muscle mass and function, which in turn has a considerable impact on the risk of falls and the ability to perform activities of daily living, thus affecting the quality of life (Jendricke et al., 2019; Vera et al., 2024; Tian et al., 2024). In fitness and bodybuilding training, which entails the activation of spinal muscles and, consequently, recruiting all body muscle groups, balance, strength, and conditioning-oriented exercises are particularly important (Iljınaite et al., 2024). In this type of exercise, multiple muscle groups are activated in a coordinated manner, thereby stimulating the nervous system and improving overall performance. This approach is particularly effective for enhancing functional strength and endurance (Oliver and Di Brezzo, 2009).

Just as training in many sports branches affects athletes, fitness and bodybuilding athletes are also physically affected by long-term and continuous training. Since it is a branch where aesthetic appearance concerns are prioritized, the effects of weight training on physical appearance must be examined. Although decreasing fat mass and increasing muscle ratio is important for looking healthy and fit, it is insufficient for aesthetic appearance. The skeletal system should function properly to contribute to the overall body appearance. Since lifting and carrying weights are involved in fitness and bodybuilding training, the load on the spinal cord increases. Structural disorders in the spinal cord are generally kyphosis, lordosis, and scoliosis. There is no clear information in the literature about the positive or negative effects of long-term weight training on the spinal cord system. Therefore, this study was designed with the hypothesis that the changes in spinal posture of individuals who regularly perform fitness and bodybuilding training will show significant differences compared to individuals who do not engage in sports, with fitness and bodybuilding trainees expected to exhibit greater thoracic kyphosis, lumbar lordosis, and sacral kyphosis values. Additionally, the objective of the correlation analysis in this study was to investigate the relationships between spinal posture parameters (such as thoracic kyphosis, lumbar lordosis, and sacral kyphosis) and the duration of fitness and bodybuilding training. This analysis aimed to explore whether the duration of training is associated with spinal curvatures. Additionally, the hypothesis of this correlation analysis is that a positive relationship is expected between the duration of fitness and bodybuilding training and spinal posture parameters (thoracic kyphosis, lumbar lordosis, and sacral kyphosis). It is believed that longer training durations, especially those involving heavy loads and repetitive postural adaptations, may lead to specific changes in spinal curvatures.

## 2 Material and methods

### 2.1 Participants

A total of 252 male individuals between the ages of 20–28 (mean age: 23.71 ± 1.81 years), who either engage in fitness and bodybuilding training (athletes) (n = 125; age 23.61 ± 1.78 years; sports age 5 ± 0.89 years) and those who do not engage in sports (sedentary) (n = 127; age 23.82 ± 1.83 years participated in the study. The athlete group consisted of individuals who regularly trained in fitness and bodybuilding for at least 4 years and exercised at least 3 times a week. The sedentary group consisted of individuals who do not engage in regular exercise. Participants should not have any spinal or joint disorders (such as osteoporosis, scoliosis, etc.). Additionally, individuals undergoing physical therapy or rehabilitation and those who had exercised on the day of measurement were excluded from the study. This study design is a cross-sectional study. G*power 3.1.9.2 software (Germany) was employed to determine the sample size. The sample size was determined using a medium effect size of 0.40, a statistical significance level of 0.05, and a high statistical power of 0.95, showing that 70 subjects were sufficient. All participants were informed about the experimental procedures and the aim of the study before providing their written informed consent. The study was approved by the 2022/47 ethics committee decision of the Ondokuz Mayıs University Clinical Research Ethics Committee and conducted in accordance with the Declaration of Helsinki.

### 2.2 Spine analysis

Spinal Mouse, a radiation-free device frequently used in the clinical evaluation of the vertebral column, was used in this study. The device can evaluate the spine in two different planes, sagittal and frontal, in three different positions: standing flexion, extension, and standing upright. In this study, measurements were taken only in an upright position. In the measurements, all spinal processes from C7 to S1 were evaluated. Data was collected by moving the Spinal Mouse along the spinous processes at a constant speed. The device transmitted the data collected every 1.3 mm to the computer at a sampling rate of approximately 150 Hz. In the measurements, thoracic kyphosis, lumbar lordosis, sacral kyphosis angle values, and sagittal upright posture of the entire spine were evaluated. With the results obtained, thoracic curvature (from T1-2 to T11-12), lumbar curvature (from L1 to L5), sacral curvature (from S1 to S5), and angular upright position of the entire spine was determined.

### 2.3 Statistical analysis

Statistical analysis of the data obtained in the study was made using the SPSS 21 package program. Data were checked for normality using the Kolmogorov-Smirnov test. Accordingly, the data was analyzed by the Independent-Samples T-test and Pearson correlation test. Statistical significance was accepted as p < 0.05.

## 3 Results

Table 1 presents the average age of all participants. The mean age was 23.71 ± 1.81 years in all participants. Athletes’ age was 23.61 ± 1.78 years while their sports age was 5 ± 0.89 years. In the control group, the mean age was 23.82 ± 1.83 years.

**TABLE 1:** Descriptive information of participants.

		n	Mean	SD
Athletes	Age (years)	125	23.61	1.78
	Sports Age (years)		5.00	0.89
Controls	Age (years)	127	23.82	1.83
Total	Age (years)	252	23.71	1.80

**FIGURE 1:**
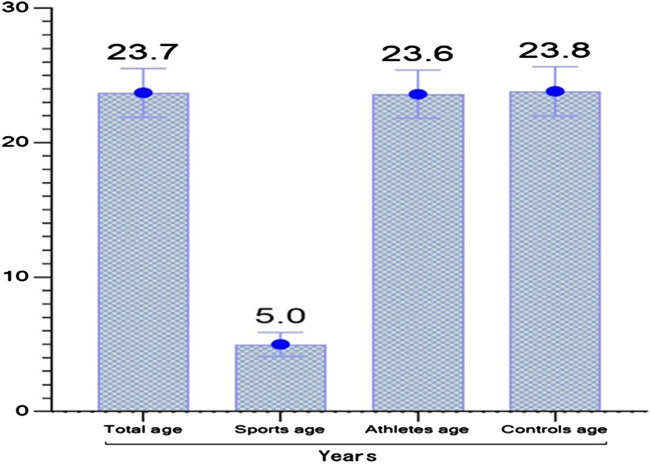
Descriptive information of participants.

Figure 1 shows the average age of all participants. In addition, the age, sports age of the fitness and bodybuilding training group, and the average age of the sedentary group are presented. The mean age of the participants engaged in fitness and bodybuilding training was 5 years.

**FIGURE 2:**
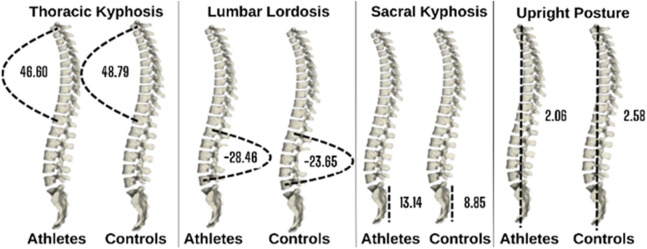
Spine values of athletes and sedentary.

Spine values of athletes and sedentary individuals are presented in Figure 2. While the athletes' thoracic kyphosis angle was 46.60, their lumbar lordosis angle was −28.46, their sacral kyphosis angle was 13.14 and their upright posture value was 2.06; the thoracic kyphosis angle of sedentary individuals was 48.79, the lumbar lordosis angle was −23.65, the sacral kyphosis angle was 8.85 and the upright posture value was 2.58.

Table 2 presents spine values and athletes and control (sedentary individuals) groups. While the athletes' thoracic kyphosis angle was 46.60, their lumbar lordosis angle was −28.46, their sacral kyphosis angle was 13.14 and their upright posture value was 2.06; the thoracic kyphosis angle of sedentary individuals was 48.79, the lumbar lordosis angle was −23.65, the sacral kyphosis angle was 8.85 and the upright posture value was 2.58.

**TABLE 2:** Comparison of spine values of athletes and sedentary individuals.

	Group	n	Mean	SD	p	Effect size
Thoracic kyphosis	Athletes	125	46.60	9.641	0.047	0.25
	Controls	127	48.79	7.641		
Lumbar lordosis	Athletes	125	−28.46	8.252	0.001	0.62
	Controls	127	−23.65	8.351		
Sacral kyphosis	Athletes	125	13.14	5.991	0.001	0.65
	Controls	127	8.85	7.111		
Upright position	Athletes	125	2.06	2.539	0.082	0.22
	Controls	127	2.58	2.162		

**FIGURE 3:**
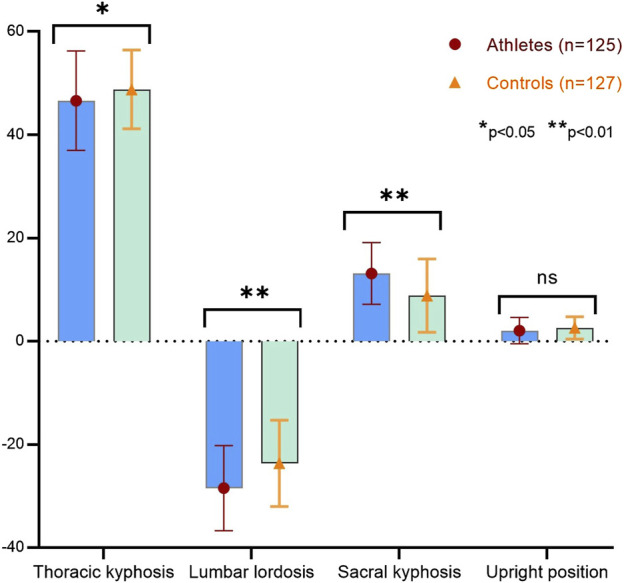
Comparison of spine values of athletes and sedentary individuals.

In Figure 3, a comparison of the thoracic kyphosis, lumbar lordosis, sacral kyphosis, and upright position values of both athlete and sedentary groups are presented. When the athlete group was compared with the sedentary group, a statistically significant difference was detected in thoracic kyphosis (p < 0.05), lumbar lordosis, and sacral kyphosis (p < 0.01) values, while no significant difference was detected in upright position values (p > 0.05).

Table 3 presents the correlation between the ages of all participants and their thoracic kyphosis, lumbar lordosis, sacral kyphosis, and upright posture. It was determined that there was no statistically significant relationship between age and the values of thoracic kyphosis, lumbar lordosis, sacral kyphosis, and upright posture (p > 0.05).

**TABLE 3:** Correlation between the age of all participants and spine values.

		Thoracic kyphosis	Lumbar lordosis	Sacral kyphosis	Upright position
Age	r	−0.031	0.037	−0.017	0.093
	p	0.623	0.554	0.787	0.142
	n	252	252	252	252

Table 4 presents the correlation between sports ages and thoracic kyphosis, lumbar lordosis, sacral kyphosis, and upright postures of the athlete group. It was determined that there was no statistically significant relationship between sports age and the values of thoracic kyphosis, lumbar lordosis, sacral kyphosis, and upright posture (p > 0.05).

**TABLE 4:** Correlation between sports age and spine values of individuals doing fitness and bodybuilding.

		Thoracic kyphosis	Lumbar lordosis	Sacral kyphosis	Upright position
Sports Age	r	0.101	−0.035	−0.050	−0.154
	p	0.264	0.697	0.580	0.087
	n	125	125	125	125

## 4 Discussion

The aim of this study was to examine the changes in the spinal postures of individuals who regularly engage in fitness and bodybuilding training. To our knowledge, there is no conclusive evidence in the literature regarding the long-term effects of fitness and bodybuilding training on the spinal cord system, whether beneficial or detrimental. With regular and intensive exercise, coordination between the spine, disks, ligaments, and muscles may deteriorate. This can affect the degree of physiological curvature of the spine, leading to a deterioration in the quality of posture. Changes in the structural balance of the spine can cause both postural problems and movement restrictions, which in the long term can negatively affect athletes' performance and overall health (Lichota et al., 2011). On the other hand, participation in long-term sports events and training load can lead to functional and structural adaptations in the athlete’s sport-specific body structure (Sanz-Mengibar et al., 2018). The main finding of this study was that athletes have better postural characteristics in thoracic kyphosis, lumbar lordosis, and sacral kyphosis compared to sedentary individuals, while no statistically significant relationship was found between age or sports age and these values.

In our study, the thoracic kyphosis and lumbar lordosis values in the upright position of athletes were lower than sedentary, while the sacral kyphosis values of athletes were higher than sedentary, these differences in our study reached statistical significance. No statistically significant difference was found between the groups in the average values of the upright position. The underlying mechanism for this is that regular and intensive training impairs the adaptive capacity of the passive structures in the spine and the active structures, namely, the muscles. Continuous high-intensity exercises can reduce the adaptive abilities of the muscles and may impair the movement mechanics and stability of the spine. This lack of adaptation can interfere with the spine’s natural functioning, leading to postural deformities and potential injuries (Huang et al., 2022). In sports disciplines where repetitive postures dominate during the training, sport-specific adaptations develop in the spine. This is associated with the observation that athletes frequently employ postural characteristics result in substantial structural alterations to the spine. Sports-specific postural tendencies affect the structural properties of the spine and develop optimal adaptations for the athlete’s performance. This adaptation provides the theoretical foundation for the distinctive postural variations observed in certain sports (de Baranda et al., 2020). Accordingly, given that bodybuilding training is typically performed at submaximal levels, it can be suggested that the results of the present study are consistent with the literature. Indeed, in clinical postural assessments, angles between 20 and 40 were normal for thoracic kyphosis, while angles between 20 and 45 were normal for lumbar lordosis, but this condition is generally used to evaluate sedentary individuals (Gonzalez-Galvez et al., 2019). The results obtained in both most sports disciplines and our study were found to be above physiological limits. This shows that it is integrated into the body’s needs and the load it is exposed to (Prêteux et al., 1985).

No study has been found in the literature, especially with individuals engaged in bodybuilding and fitness training, so our results were compared with similar studies conducted with athletes from different disciplines (rock climbing, cycling, tennis, rowing, wrestling). Förster et al. (2009) in their study with elite male rock climbers, determined that the thoracic kyphosis angle of the rock climbers was 48.48° and the lumbar lordosis angle was −28.98°. In their study, Muyor et al. (2011) found that the thoracic kyphosis angle of elite cyclists was 48.17, the lumbar lordosis angle was −27.3, and the sacral kyphosis angle was 11.25. Also, the same researchers observed the thoracic kyphosis angle to be 43.83 and the lumbar lordosis angle to be −27.58 in elite male tennis players (Muyor et al., 2013). López-Miñarro et al. (2010) in their study with 40 elite young rowers aged 15–16, reported that the thoracic kyphosis angle was 45.53° and the lumbar lordosis angle was −28.23°. Rajabi et al. (2008) found a statistically significant difference in a study comparing the thoracic kyphosis degrees of freestyle (30°), Greco-Roman (24.3°), and sedentary (27.4°) individuals. Kielt et al. (2014), in their study on two groups of rock climbers and non-rock climbers, found the thoracic kyphosis value of climbers to be 47.65, the lumbar lordosis value to be −21.11, and the thoracic kyphosis value to be 28.94 and the lumbar lordosis value to −19.65 in sedentary individuals. In the present study, it can be suggested that fitness and bodybuilding training are congruent with the results obtained in other sports regarding thoracic kyphosis and lumbar lordosis angle values. Furthermore, no evidence supports the assumption that such training impairs the degree of thoracic kyphosis and lumbar lordosis.

In the study examining different sports and training duration, it was reported that thoracic kyphosis increased proportionally with the annual training period (hours/year), but there was no change in lumbar lordosis until the training period exceeded 400 h/year. In addition, the researchers reported a relationship between the increase in thoracic kyphosis and lumbar lordosis angles and high-intensity training. It was observed that the risk of developing excessive kyphosis was higher in children who performed high-intensity sports than in children who did not. Moreover, study results indicated that lack of physical activity may also contribute to the development of kyphosis in healthy children. These findings demonstrate the negative effects of excessive sports and insufficient physical activity on spinal health (Wojtys et al., 2000). In their study on wrestling, gymnastics, football, and tennis athletes and sedentary individuals, Hellstrom et al. (1990) reported that abnormalities in the vertebral ring apophysis were observed only in athletes, however, in the group that trained regularly, there was a decrease in disc height due to exposure to more load than normal.

In the current study, no statistically significant correlation was found between the age of all participants and the sports age of individuals who engage in fitness and bodybuilding training and thoracic kyphosis, lumbar lordosis, sacral kyphosis, and upright posture values. In the literature, Porter et al. (1989) reported that the columna vertebralis changes physically and physiologically due to age and the loads, and it is constantly trying to renew itself against external loads. However, considering the average age (23 years) and sports age of the participants in this study, it can be speculated that adequate training volume could not be reached. Regarding the average sports age (5 years), this period seems insufficient for physical and physiological changes.

The present study results indicated that intensive training with high loads should be avoided at a young age, especially before adolescence, to avoid problems in spinal posture and health. Considering the effects of sport-specific movements on physical posture, getting technical training and applying the movements correctly is necessary to prevent posture deformities that may continue for a long time. Long-term and regular training has significant effects on the muscles, which are the active elements of the spine. Therefore, stretching the muscles is critical to avoid these negative effects. Especially in the pre-adolescence period, appropriate adjustment of the training load and modalities regarding the characteristics of the different sports is valuable in preventing postural deformities.

The present study has several limitationsInitially, the study population was restricted to males aged 20–28 years. This may limit the generalizability of the study’s findings to other age groups, including women. Additionally, the study did not consider other factors, such as the intensity of physical activity. Therefore, further research is necessary to elucidate the observed phenomena’s full scope and extend the findings to a more diverse population. In addition, the study focused on specific spinal curvatures, and other components of posture, including balance and flexibility, were not assessed. Furthermore, the participation of athletes in other sports or physical activities and the intensity of these activities were not considered. Therefore, future studies should include a larger demographic group and employ different posture assessments.

## 5 Conclusion

In conclusion, the results suggested that individuals engaging in fitness and bodybuilding training showed significant differences in certain spinal curves. However, these values remained within physiological limits and did not indicate any negative effects. This study contributes to the understanding of the impact of fitness and bodybuilding training on spinal health, emphasizing the importance of regular exercise in maintaining optimal posture and preventing spinal disorders.

## Data Availability

The raw data supporting the conclusions of this article will be made available by the authors, without undue reservation.
